# Public Awareness and Perceptions of Colorectal Cancer Prevention: a Cross-Sectional Survey

**DOI:** 10.1007/s13187-020-01721-5

**Published:** 2020-02-28

**Authors:** Markus Dines Knudsen, Geir Hoff, Ida Tidemann-Andersen, Gry Ekeberg Bodin, Sissel Øvervold, Paula Berstad

**Affiliations:** 1 Section of Bowel Cancer Screening, Cancer Registry of Norway, Oslo, Norway; 2 Department of Research and Development, Telemark Hospital, Skien, Norway; 3 The Norwegian Cancer Society, P.O. Box 4, Centrum, 0101 Oslo, Norway; 4 Department of Health Management and Health Economics, University of Oslo, Oslo, Norway; 5 Present Address: Western Norway Research Institute, Sogndal, Norway

**Keywords:** Colorectal cancer, Prevention, Screening, Risk factor, Lifestyle, Awareness

## Abstract

We aimed to investigate awareness of colorectal cancer (CRC) lifestyle risk factors, willingness to participate in CRC screening, and preferences concerning channels for information on CRC prevention in the general population, including the target age of the upcoming Norwegian national CRC screening program. The present study was a cross-sectional online survey of adults aged 39 to 55 years registered as Kantar Web Panel respondents in Norway. The survey included demographic characteristics, multiple choice knowledge questions of lifestyle risk factors for CRC, attitudes towards CRC screening, and preferred channels for receiving information on CRC prevention. Of 4375 participants invited, 2007 (46%) answered the survey. The average number of correctly identified lifestyle risk factors for CRC was 7.3 of ten. Women were significantly more likely than men, and those with university or college education more likely than those with lower education to correctly identify at least eight risk factors (odds ratio, OR = 1.53, 95% confidence interval, CI 1.25–1.87, and OR = 1.51, 95% CI 1.23–1.86, respectively). The number of correctly identified risk factors was positively associated with willingness to participate in CRC screening (*P* for trend < 0.001). The national public work force and the Norwegian Cancer Society were selected by 76% and 69% of the participants, respectively, to be trustworthy sources of information on CRC prevention. Awareness of CRC risk factors was associated with willingness to participate in CRC screening. The national public work force and Cancer Society can be generally accepted sources of CRC preventive information.

## Introduction

The importance of healthy lifestyle behaviors has been acknowledged in international cancer prevention recommendations [1] and strategies [2]. Colorectal cancer (CRC) is one of the cancers taking most lives and is largely preventable by physically active lifestyle, normal body weight, healthy diet, and by avoiding alcohol consumption and smoking [1, 3]. Public strategies to promote favorable behaviors may have a large potential to prevent CRC. Incidence of CRC is high in Norway [4]. A national CRC screening program is being planned, and will be gradually rolled out in Norway from 2021 on. Strategies targeted to improve CRC preventive lifestyles in the general population might strengthen a national effort to reduce CRC mortality.

For increasing public awareness of health behaviors, carefully conducted mass media campaigns may have an effect [5, 6]. In Norway, a number of mass media campaigns to improve health behaviors have been arranged by the Directory of Health which is the national public health workforce, but also by nonprofit organizations as well as commercial actors. Free digital aids such as applications for smoking cessation are available. However, in the lack of evaluation of effect on awareness or health behavior changes in most of the campaigns, their impact on public health cannot be concluded. Interventions to successfully improve lifestyle behavior in the general population require large resources [7–9]; otherwise, the effect is limited [10]. Targeted attempts, e.g., engaging in specific arenas and through selected information channels, may be best suited to reach high-risk lifestyle individuals.

A CRC screening program may serve as a feasible setting to increase public awareness on lifestyle risk factors [11–14]. For developing the most suitable information strategies, it is useful to investigate the existing knowledge of CRC prevention as well as the preferred channels for information in the target population. Attitudes for CRC screening and cancer-preventing information are still unknown in the future screening target population in Norway. When the national CRC screening program in Norway will be fully implemented, each 55-year-old age cohort of approximately 75,000 individuals will be invited to five biennial rounds of immunochemical fecal occult blood testing (iFOBT). In the currently running piloting of the national screening program in selected municipalities [15], uptake in accumulated three iFOBT rounds is 68%.

Our aim was to investigate willingness to participate in CRC screening and awareness of CRC lifestyle risk factors in the general population in the age group of the upcoming Norwegian national CRC screening program, as well as in younger adults who will reach the screening age during the next 16 years. We also aimed to explore preferences concerning channels for information on CRC prevention in this population.

## Methods

### Study Population

The survey was administrated by the data collection specialist Kantar, which recruits panelists through numerous websites and telephone calls, and invites them to participate in online surveys in exchange for small financial rewards. The financial reward for participating in the present survey was eight Norwegian kroner, equivalent to some less than one American dollar. The survey was carried out from the beginning of May till the end of June 2018. The survey was sent to a random selection of 4375 men and women aged 39 to 55 years, registered in Kantar Web Panel’s database in Norway. The oldest individuals in this selection represent birth cohorts that will be invited to the upcoming national Norwegian CRC screening when successively reaching the screening age of 55 years. Younger birth cohorts were included as they are about to reach the age at which cancer is becoming more prevalent but preventive lifestyle changes still may be effective. The survey was available only online. Participants’ age, gender, education, ethnicity, area of residence, and household’s income were available from the Kantar’s database. When signing up for Kantar Web Panel, the participants gave their informed consent for their data to be used for scientific research. Data was anonymized for the researchers. Because anonymous data was used, there is no requirement of approval by research ethical committee according to the Norwegian health research law § 4-d [16].

### Questionnaire

In the survey, all questions were supposed to be responded by closed-ended answering options. The participants were asked “How do you evaluate your own knowledge on how to reduce your risk for CRC?” with the options of “very large,” “large,” “neither,” “small,” “very small,” or “do not know.” They were then asked to estimate the association with the following lifestyle factors and CRC risk: alcohol, processed meat, red meat, fish, fruit and vegetables, wholegrain products, dairy products, chocolate, sugar, cod liver oil, physical activity, smoking, in addition to overweight. The answering options were as follows: “decreases the risk,” “does not affect the risk,” and “increases the risk.” It was further asked “Would you like to get information on how to reduce your risk of CRC” and “In what way would you prefer to receive information on how to reduce your risk of CRC,” followed by “If you were to get information and advice on how to reduce your risk of CRC, who would be a trustworthy sender?”

The individuals were also asked whether they know what cancer screening is and how likely they were to participate in the upcoming national Norwegian CRC screening program. Alternative reasons for likely or unlikely participation were given. Lastly, the participants were asked if they found it difficult to know which health recommendations to follow with the alternative answers “agree,” “disagree,” or “neither.”

### Categorization of Variables

Participant’s evaluation of his/her own knowledge on how to reduce the risk for CRC was categorized as “large,” “neither/do not know,” or “small.” The knowledge questions on the association between different lifestyle factors and CRC risk were dichotomized to correct or incorrect answer. The correct answers were as follows: alcohol, processed meat, red meat, overweight, and smoking increase the risk of CRC; physical activity, wholegrain, fish, fruit and vegetables, and dairy products decrease the risk of CRC while chocolate, sugar, and cod liver oil do not affect the risk. Based on the ten risk increasing and decreasing factors, we created a knowledge score of CRC risk factors (range from zero to ten points), giving one point for each correct answer. We further categorized the participants into those who correctly identified eight or more risk factors (score ≥ 8) and less than eight (score < 8), used in the analyses in which knowledge was the outcome variable.

### Statistics

Descriptive statistics included number and percentage. Logistic regression analyses were used for binary variables. Predictor variables for knowledge of lifestyle risk factors and for preferences of the source of information were the demographic characteristics: sex, age group, household’s income, and educational level. Outcome variables were the knowledge score of lifestyle factors associated to CRC risk and the information source choices selected by the participant. However, in the analysis of the association between the knowledge of risk factors and willingness to participate CRC screening, the knowledge score was the predictor, and willingness to participate (yes/no/do not know) was the outcome. Likeliness of correct response, preferences for information source, and willingness to participate in CRC screening in subgroups compared with a reference group are presented by odds ratios (OR) with 95% confidence intervals (CI). The regression analyses were adjusted for gender, age (39–44 years, 45–55 years), area of residence (capital area of Oslo and Akershus counties, or Eastern, Southern, Western, central or Northern Norway), occupation (full time employed, part time employed, unemployed/social insurance, other), household’s income in Norwegian kroner (< 800,000, ≥ 800,000), and educational level (lower than university/college, university/college degree). Individuals with any missing value were categorized as missing for that particular variable in the regression model. All analyses were also conducted in the subgroup of 51–55-year-old respondents, which is the age group soon to be invited to the national CRC screening program. We performed the statistical analyses as complete case analyses using the STATA™ software, version 14.2 (Stata Corp, College Station, TX, USA).

## Results

Out of the 4375 invited, 2007 (46%) completed the survey. The respondents’ sociodemographic characteristics are shown in Table 1. The mean age was 47.4 years; 50% were women and 63% had a university/college education. Fifty-nine percent had a household income of > 800,000 NOK. Thirty-eight percent were from Eastern Norway and 98% had full time work. The number of participants with missing background information was 228 (11%) for ethnicity and 113 (6%) for household income (Table 1).

**Table 1:** Characteristics of the study population

	Total, *n* = 2007 *n* (%)	Male, *n* = 1007 *n* (%)	Female, *n* = 1000 *n* (%)	*P* value
Age (years)				0.017
30–44	725 (36)	338 (34)	387 (39)	
45–59	1282 (64)	669 (66)	613 (61)	
Ethnicity				0.192
Norwegian	1593 (79)	800 (79)	793 (79)	
Other	186 (9)	102 (10)	84 (8)	
Missing	228 (11)	123 (12)	105 (11)	
Educational level				< 0.001
Primary school (7–10 years)	93 (5)	46 (5)	47 (5)	
High school (general)	181 (9)	86 (9)	95 (10)	
High school (vocational)	295 (15)	163 (16)	132 (13)	
Technical	178 (9)	114 (11)	64 (6)	
University/college (minimum 4 years)	1260 (63)	598 (59)	662 (66)	
Household income (Norwegian kroner)				< 0.001
< 200,000–400,000	125 (7)	44 (5)	81 (9)	
400,000–800,000	515 (28)	246 (27)	269 (29)	
800,000–1,200,000	697 (38)	364 (40)	333 (36)	
More than 1,200,000	388 (21)	218 (24)	170 (19)	
Missing	113 (6)	45 (5)	68 (7)	
Area of residency				0.508
Capital area Oslo/Akershus	442 (22)	222 (22)	220 (22)	
Eastern Norway	767 (38)	384 (38)	384 (38)	
Southern and Western Norway	301 (15)	162 (16)	139 (14)	
Central and Northern Norway	497 (25)	240 (24)	257 (26)	
Occupation				< 0.001
Full time work	1595 (80)	882 (88)	713 (71)	
Part time work	201 (10)	39 (4)	162 (16)	
Social insurance	167 (8)	66 (7)	101 (10)	
Other^a^	44 (2)	20 (2)	24 (2)	

^a^Other = at home, student, retired

Respondents’ knowledge about and attitudes towards CRC screening and health recommendations are shown in Table 2. What cancer screening is was known by 46% of the responders. After having been presented a text describing stool-based CRC screening method, 87% of the respondents were likely to participate in a future national CRC screening program. This proportion was 89% in the age group of 51–55 years (results not shown). The most preferred reason for being positive to participation was early detection, selected by 91%. Thirteen percent answered that they were unlikely or neither likely/unlikely to participate in CRC screening. Of these, 19% assumed that the screening method would be uncomfortable. Forty-four percent found it difficult to know which health recommendations to follow. Individuals with a university/college degree were significantly less likely to give this response than lower educated individuals (OR 0.41, 95% CI 0.25–0.65, results not shown). Fifty-four percent evaluated their knowledge on how to reduce their risk for CRC as small (Table 2). Sixty-eight percent of the respondents were positive to receiving information on how to reduce their risk of CRC (Table 2).

**Table 2:** Knowledge about and attitudes towards colorectal cancer screening and health recommendations

	Total, *n* = 2007 *n* (%)	Men, *n* = 1007 *n* (%)	Women, *n* = 1000 *n* (%)	*P* value
Do you know what cancer screening is?				< 0.001
Yes	921 (46)	393 (39)	528 (53)	
No/do not know	1079 (54)	612 (61)	467 (47)	
Missing	7 (0)	2 (0)	5 (1)	
How likely are you to attend a national CRC screening program using fecal immunochemical test				0.035
Likely	1744 (87)	857 (85)	887 (89)	
Neither	174 (9)	101 (10)	73 (7)	
Unlikely	82 (4)	47 (5)	35 (4)	
Missing	7 (0)	2 (0)	5 (1)	
Why are you *likely* to attend a national CRC screening program? (*n* = 1744)				All > 0.05
Early detection	1594 (91)	784 (92)	810 (91)	
Know someone who has had CRC	267 (15)	118 (14)	149 (17)	
Trust the government’s recommendations	257 (15)	133 (16)	124 (14)	
Other	38 (2)	15 (2)	23 (3)	
Do not know	1 (0)			
Missing				
Why are you *unlikely* to attend a national CRC screening program? (*n* = 197)				All > 0.05
Struggle with other health challenges	25 (13)	16 (14)	9 (11)	
Do not believe in screening	25 (13)	17 (15)	8 (10)	
The screening method is disgusting	20 (10)	10 (9)	10 (13)	
The screening method is uncomfortable	38 (19)	23 (20)	15 (19)	
Limited time	14 (7)	11 (9)	3 (4)	
Other	42 (21)	23 (19)	19 (24)	
Do not know	45 (23)	26 (22)	19 (24)	
How do you evaluate your knowledge about how to reduce your own risk of CRC				< 0.001
Large	318 (16)	111 (11)	207 (21)	
Neither/do not know	610 (30)	301 (30)	309 (31)	
Small	1075 (54)	594 (59)	481 (48)	
Missing	4 (0)	1 (0)	3 (0)	
Would you like to get information on how to reduce your risk of CRC?				0.404
Yes	1368 (68)	693 (69)	675 (68)	
No/do not know	627 (31)	305 (30)	322 (32)	
Missing	12 (1)	9 (1)	3 (0)	
I find it difficult to know which health recommendations to follow				0.008
Agree	916 (46)	464 (46)	452 (45)	
Disagree	585 (29)	265 (26)	320 (32)	
Neither	492 (25)	269 (27)	223 (22)	
Missing	14 (1)	9 (1)	5 (1)	

*CRC* colorectal cancer

**Fig. 1:**
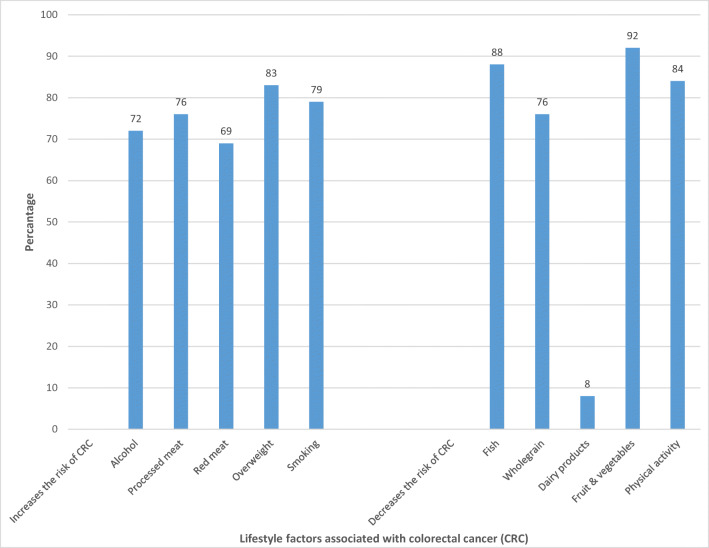
Percentage of correct answers for the association between lifestyle factors and risk of colorectal cancer

The percentage of correct answers for single factors ranged from 8% for dairy products to 92% for fruit and vegetables (Fig. 1). The mean knowledge score (range 0–10) was 7.3, and 60% of the participants correctly identified at least eight of ten CRC lifestyle risk factors.

The adjusted likelihood of correct identification of a minimum of eight risk factors in subgroups is shown in Table 3. Compared with men, women were significantly more likely to correctly identify a minimum of eight CRC lifestyle risk factors (OR 1.53, 95% CI 1.25–1.87). Compared with individuals with less education, those with a university/college degree were significantly more likely to correctly identify a minimum of eight CRC lifestyle risk factors (OR 1.51, 95% CI 1.23–1.86) (Table 3). In the subgroup of 51–55-year-old individuals, it was even more likely that the university/college educated correctly identified a minimum of eight risk factors compared with the lower educated (OR 2.36, 95% CI 1.64–3.42). There was, however, not a pronounced knowledge difference between men and women in that age group (results not shown).

**Table 3:** Demographic predictors of correct identification colorectal cancer lifestyle risk factors^a^

	Participants who correctly identified a minimum of eight of ten risk factors, *n* (%)	OR (95% CI)^b^
Gender		
Male	525 (55)	Ref.
Female	601 (63)	1.53 (1.25–1.87)
Age (years)		
30–44	393 (57)	Ref.
45–59	733 (60)	1.25 (1.01–1.53)
Household income (Norwegian kroner)		
< 800.000	338 (56)	Ref.
≥ 800.000	649 (63)	1.27 (1.02–1.57)
Educational level		
Lower than university/college	361 (52)	Ref.
University/college (minimum 4 years)	765 (63)	1.51 (1.23–1.86)

*OR* odds ratio, *CI* confidence interval

^a^Smoking, alcohol, overweight, physical activity, red meat, processed meat, fruit and vegetables, wholegrain, fish, and dairy products

^b^Likelihood of correct identification of a minimum of eight of ten colorectal cancer lifestyle risk factors adjusted for gender (male, female), age (30–44 years, 45–59 years), area of residency (capital area Oslo/Akershus, Eastern, Southern and Western, Central and Northern Norway), occupation (full time work, part time work, social insurance, other), household income in Norwegian kroner (< 800,000, ≥ 800,000), educational level (lower than university/college, university/college of minimum 4 years)

Table 4 shows the association between the CRC risk factor knowledge score and willingness to participate in a national CRC screening program. There was a significant trend (*p* < 0.001) between the knowledge score and willingness to participate in the CRC screening program. Individuals with the highest score (9–10) had an OR of 2.29 (95% CI 1.52–3.45) for intention to participate in screening compared with individuals with the lowest score (0–7) (Table 4). This trend was similar in the age group 51–55 years (results not shown).

**Table 4:** Association between the knowledge score and willingness to participate colorectal cancer screening

Score for knowledge on CRC lifestyle risk factors^b^	Total, *n* = 1849	Willing to participate in CRC screening	Absolute estimate per 100 for participating in CRC screening	OR (95% CI)^a^	*P* trend
	*n*	*n*	*n*		
0–7	748	646	86	Ref.	< 0.001
8	441	394	89	1.28 (0.87–1.90)	
9–10	660	621	94	2.29 (1.52–3.45)	

*OR* odds ratio, *CI* confidence interval, *CRC* colorectal cancer

^a^Adjusted for gender (male, female), age (30–44 years, 45–59 years), area of residency (capital area Oslo/Akershus, Eastern, Southern and Western, Central and Northern Norway), occupation (full time work, part time work, social insurance, other), household income in Norwegian kroner (< 800,000, ≥ 800,000), educational level (lower than university/college, university/college of minimum 4 years)

^b^The number of correct answers for the association between smoking, alcohol, overweight, physical activity, red meat, processed meat, fruit and vegetables, wholegrain, fish and dairy products, and risk of colorectal cancer

Directory of Health, the Norwegian Cancer Society, and general practitioners were considered as the three most trustworthy sources, selected by 76%, 69%, and 61% of the participants, respectively. Women were significantly more likely than men to select the Norwegian Cancer Society as a trustworthy information source (OR 1.76, 95% CI 1.43–2.17), but less likely than men to choose general practitioners (OR 0.81, 95% CI 0.67–0.89). In the subgroup of 51–55-year-old individuals, CRC screening program was additionally selected by 62% as a trustworthy information source (results not shown).

## Discussion

In the present survey, we observed that less than half of 39–55-year-old men and women knew what cancer screening is. Despite this, 87% considered themselves likely to attend the forthcoming national CRC screening program when being informed of the stool-based CRC screening method. The survey also showed that high knowledge of CRC risk factors was associated with intention to participate in CRC screening. Of particular interest is that we observed this association in men and women of age 51–55 years, the age group soon to be invited to CRC screening. Knowledge of these risk factors was better among women than men and, as expected, better among those with higher education. The majority of the respondents were interested in receiving information on how to reduce their risk of CRC. Preferences for trustworthy source of this information were associated with gender, educational level, and household income. However, Directory of Health and the Norwegian Cancer Society were considered as trustworthy sources by the majority.

To the best of our knowledge, this is the first study combining public attitudes towards CRC screening, awareness of lifestyle risk factors for CRC, and preferences for cancer preventive information. The mean knowledge in the present study was relatively high, 7.3 correct of ten CRC risk factors. Results from earlier UK studies showed lower knowledge level compared with our study [17, 18]. It could be due to lower educational level (42% with a university degree in the study by Lynes [17] versus 67% in the present study). In our study, educational level was associated with knowledge of CRC risk factors, as established earlier [19–21]. Difference from earlier studies in the knowledge level may also be due to a different way of assessing the knowledge (open-ended questions in the study by Anderson [18] versus given answering options in the present study). Also, the population in the study by Anderson was a selected group of overweight individuals diagnosed with adenoma. A study in 21 European countries from 2004 assessed awareness of CRC lifestyle risk factors by only three questions in a wide commercial questionnaire. That study showed that 59% of the Norwegian participants identified physical inactivity as a CRC risk factor [22]. In our study, 84% did so. The difference may be due to an increase in media coverage on CRC and risk factors after 2004 until the present study, which may have increased the public awareness.

Our results showed a clear association between the knowledge of risk factors for CRC and intention to participate in the forthcoming CRC screening program, in line with the findings from previous studies from the UK and Spain [21, 23]. It may therefore be expected that increasing public awareness of CRC prevention will increase CRC screening uptake. According to our results, the most important target populations for information are groups of low educated people, as well as men. Men preferred general practitioner as a trustworthy information source higher than women in this study, indicating preference for direct and personal advice by an expert. This can be an important issue to take into account when planning information strategies. Gender differences in our survey are supported by earlier research which shows that women are more interested in and more actively seek information of health and disease prevention [24, 25].

The present study has some important strengths. The analyses were based on a large survey with participants resident in all geographical areas in Norway. We used a detailed and comprehensive questionnaire to assess knowledge about risk factors for CRC. Timely for the ongoing planning of the national CRC screening program, the participant age group represented the future invitees of the program. However, there is a possibility that individuals with a greater interest in the topic of cancer prevention were more likely to respond to the survey. Also, the overrepresented selection of highly educated participants is a limitation of this study; 59% of men and 66% of women had a university/college degree, while 33% of men and 44% of women in the age group 40–59 years have this level of education according to Statistics Norway. Primary school was the highest education of 5% of the participants in this study, while approximately 20% of the adult Norwegian population has this education only [26]. The skewed distribution of participants according to education might have influenced our results limiting generalizability, and indicates that the actual awareness of CRC lifestyle risk factors might be lower than reported in the present survey. The online questionnaire was closed-ended and measured recognition of risk factors rather than active recall, and may therefore have given an overestimation of knowledge in the participants [27]. The lack of a validated assessment tool for CRC-related knowledge, preferences, and attitudes is limitations in both the present study, as well as earlier studies on this topic.

Although information about cancer prevention does not necessarily cause behavioral change, it may facilitate change [17] and inspire individuals at high risk towards lifestyle improvements [28]. For initiating a change in CRC preventive behaviors, CRC screening might be the most effective arena [11–14]. Importantly, the majority of the age group soon to be invited to a national CRC screening program was interested in receiving information about CRC prevention and considered CRC screening program as a trustworthy source. This finding strongly proposes advice of CRC preventive lifestyle to be included in a CRC screening program. Acceptability of lifestyle advice for CRC prevention is probably highest at CRC screening setting [28, 29]. Further investigation is needed to understand the most acceptable and effective information strategies to be implemented in a CRC screening program.

## Conclusion

The present study showed that awareness of CRC lifestyle risk factors in an age group soon eligible for CRC screening was highest in women and those with highest education. Intention to participate in the forthcoming national CRC screening program was related to awareness of risk factors for CRC. The majority in this group was interested in receiving information on CRC prevention. Effective information strategies may differ between men and women. Directory of Health, the Norwegian Cancer Society, general practitioners, and the CRC screening program were considered as trustworthy sources of information.

## Abbreviations

CI: confidence interval
CRC: colorectal cancer
OR: odds ratio
